# miR-21-5p protects IL-1β-induced human chondrocytes from degradation

**DOI:** 10.1186/s13018-019-1160-7

**Published:** 2019-05-03

**Authors:** Hai Zhu, Xin Yan, Meng Zhang, Feng Ji, Shouguo Wang

**Affiliations:** 1 Department of Orthopaedics, The Affiliated Huaian No.1 People’s Hospital of Nanjing Medical University, Huaian, Jiangsu Province China; 2 Department of Orthopaedics, Affiliated Hospital of Nantong University, Nantong, Jiangsu Province China; 3 Research Center of Clinical Medicine, Affiliated Hospital of Nantong University, Nantong, Jiangsu Province China

**Keywords:** Osteoarthritis, Chondrocytes, miR-21-5p, COL2A1, ADAMTS5, MMP13

## Abstract

**Objective:**

Osteoarthritis (OA) is a prevalent degenerative disease caused by various factors. MicroRNAs are important regulators in OA. MiR-21-5p expression is decreased in OA cartilage, but the effects of modulating miR-21-5p on cartilage regeneration are unknown. Therefore, our aim was to investigate the effects of miR-21-5p on cartilage metabolism of OA chondrocytes.

**Design:**

We used IL-1β (10 ng/ml) to mimic OA chondrocytes. OA chondrocytes were transfected with miR-21-5p, the gene expression of COL2A1, MMP13, and ADAMTS5 was detected by qPCR. At the same time, COL2A1, MMP13, and ADAMTS5 were analyzed at the protein level by Western blot. CCK8 measured the cell’s viability and SA-β-gal detected the cell’s senescence.

**Results:**

Upregulation of miR-21-5p had increased COL2A1 expression and decreased MM P13 and ADAMTS5 expression, which were in accord with Western blot data. SA-β-gal activity significantly increased, the viability was decreased in OA chondrocytes, and upregulation of miR-21-5p can decrease the SA-β-gal activity and increase cell viability.

**Conclusion:**

MiR-21-5p might be a potential disease-modifying compound in OA, as it promotes hyaline cartilage production. These results provided that novel insights into the important function in OA pathological development.

## Introduction

Osteoarthritis (OA) is the most prevalent chronic degenerative joint disease in the aging population, with patients often suffering from severe pain and limited movement, and speculated to be the greatest cause of disability in the general population by 2030 [1, 2], characterized by synovial inflammation, articular cartilage degradation, and subchondral bone remodeling. Factors that increase the susceptibility to OA include aging, obesity, genetics, and joint injury. No disease-modifying drug is currently available and no major drug target has been identified [3]. Treatment for OA remains unsatisfactory. There is a need for further research into understanding the pathogenesis and mechanisms of OA in order to identify new therapeutic targets reducing the symptoms and preventing disability.

The dysregulation of chondrocytes occurs in the pathogenesis of OA [4, 5]. Chondrocytes are the dominant cells in the articular cartilage and play an important role in the homeostasis of cartilage metabolism as well as integrity of the extracellular matrix. Loss of chondrocyte cellularity within the articular cartilage is one of the prominent events that contribute to its degradation. However, the exact molecular mechanisms that lead to the induction and progression of OA have yet to be elucidated, and completely effective therapeutic measures have not been developed. Treatment remains associated with side effects and aims to improve daily functioning and reduce symptoms, such as functional gastrointestinal disorders, providing relief from pain, and not a cure [6]. The death of chondrocytes and the loss of the extracellular matrix are the central features in cartilage degeneration during OA pathogenesis [7].

Interleukin-1β (IL-1β), a key pro-inflammatory factor, is one of the mediators of OA [8]. So in order to mimic OA condition, IL-1β was used to induce chondrocytes apoptosis in vitro. IL-1β stimulation enhances the degeneration of cartilage matrix by upregulating matrix degrading enzymes and downregulating chondrocyte-specific proteins. Our previous study confirmed that IL-1β stimulated articular normal chondrocytes to mimic OA chondrocytes [8].

It has emerged in recent years that microRNAs are involved in the onset and development of OA and can modulate various cellular processes, such as apoptosis, cell differentiation and proliferation, and matrix remodeling [9]. Emerging evidence suggests that dysregulated miRNAs play a pivotal role in OA. miRNAs are small, 18–24 nucleotides in length, single-stranded non-coding RNA molecules that can negatively regulate the expression of target genes in a post-transcriptional manner by binding to specific sequences within target mRNAs [10]. Recent studies have shown that miRNAs play a crucial role in human disease and can be a potential new therapeutic target [11]. An increasing number of reports have demonstrated that miRNAs regulate chondrogenesis and cartilage development. Profiling research suggests that miRNAs are aberrantly expressed in OA, and these may be implicated in the regulation of cartilage integrity through inflammation, cellular communication, or cell death [12].

miR-21 has been highlighted due to its importance in tumor progression and metastasis, specifically in the process of cell proliferation and differentiation, which is vital to chondrogenesis and cartilage remodeling [13–15]. However, little is known about the function of miR-21 in OA.

Progressive cartilage degradation is a hallmark of OA and is believed to be largely mediated by proteases belonging to the matrix metalloproteinase (MMP) and a disintegrin and metalloproteinase with thrombospondin motifs (ADAMTS) family of enzymes [16], both have been regarded as the most likely candidates for OA pathogenesis [17]. The deletion of ADAMTS5 in mice with OA can prevent early aggrecan depletion and cartilage destruction [18]. MMP-13 concentration can reflect the development and severity of OA over time, indicating that MMP-13 may be a promising target for treating OA. MMP13 is the main enzyme involved in cartilage collagen degradation. ADAMTS5 is the major protease responsible for cartilage degradation in osteoarthritis. MMP13 and ADAMTS5 play critical roles in cartilage extracellular matrix degradation during the pathogenesis of OA since they are responsible for the degradation of COL2A1 and proteoglycans in articular cartilage.

In the present study, we found that miR-21-5p was significantly downregulated in OA chondrocyte. According to our findings, we attempt to provide further insight into the role of miR-21-5p in the pathogenesis of OA.

## Materials and methods

### Materials

Dulbecco’s Modified Eagle Medium was obtained from Corning. Penicillin, streptomycin, and fetal bovine serum (FBS) were obtained from Gibco BRL (Grand Island, NY, USA). Recombinant human IL-1β was obtained from R&D Systems (Minneapolis, MN). Annexin V-FITC/propidium iodide (Annexin V-FIFC/PI) apoptosis kit was from BD Biosciences. A CCK8 kit was purchased from Dojindo (Kumamoto, Japan). The antibodies used in this study are as follow: ADAMTS5 from Cell Signaling Technology (Danvers, MA) and β-actin from Abcam (Cambridge, UK). Predesigned primers for MMP13, ADAMTS5, COL2A1, and GAPDH were obtained from Biomics Biotechnologies (Nantong, China). miR-21-5p primers (forward, 5′-TAGCTTATCAGACTGATGTTGA-3′ and reverse, 5′-AGTGCGTGTCGTGG-3′, Biomics, Nantong, China)

### Cell culture

The normal human articular cartilages from 6 trauma patients with mean 46.8 years (age mean, 28-59 years) were incubated with trypsin (Hyclone) at 37 °C for 10 min. After the trypsin solution was removed, the tissue slices were treated for 16 h with typeIIcollagenase (Sigma-Aldrich, Shanghai, China) in DMEM with 10% FBS. The isolated chondrocytes were recovered in DMEM supplemented with 10% FBS, 50 U/ml penicillin and 50 mg/ml streptomycin. The cells were incubated at 37 °C in the presence of 5% CO_2_. In order to mimic OA chondrocytes, we used IL-1β (10 ng/ml) to stimulate.

### Cell transfection

Chondrocytes were seeded at a density of 5 × 10^5^ cells/well in 6-well culture plates (Corning, NY, USA). The cells were transfected with miR-21 mimic (30 nM), inhibitor (50 nM), and corresponding to respective NC when the cell fusion rate reached 70% using Lipofectamine 2000 (Invitrogen, CA, USA), according to the manufacturer’s instructions. After 4 h, the transfection medium was discarded. Cells were washed with serum-free DMEM, then cultured in DMEM supplemented with 10% FBS. Forty-eight hours after transfection, the cells were treated with IL-1β (10 ng/ml) for 24 h, and then harvested for further studies.

### Cell proliferation assay

Cell proliferation was assayed by the Cell Counting Kit-8 (CCK8) assay (Dojindo) according to the manufacturer’s protocol. A total of approximately 5 × 10^3^ transfected cells were plated in 96-well plates in triplicate and cultured in 90 mL medium containing 10% FBS. Then 10 μL CCK8 solution was added to each well and incubated for 2 hours at 37 °C at each indicated time point. Absorbance was then recorded at 450 nm using the microplate reader (Japan Interned, Japan). Each experiment was repeated three times, and the data represent the mean of all measurements. The cell proliferation curves were plotted using the absorbance at each time point.

### Apoptosis analysis

Cell apoptosis were measured using an Annexin V-FITC/PI apoptosis kit. Briefly, at 48 hours post-transfection, cells were cultured with IL-1β (10 ng/ml) for 24 h and then cells were harvested by trypsinization and washed with PBS twice. Apoptotic cells were evaluated by double staining with Annexin-FITC and PI in binding buffer using by flow cytometry (Becton Dickinson). Then the cells were resuspended in binding buffer and stained with Annexin V and PI (Annexin V-FITC Apoptosis Detection Kit, eBioscience) for 15 min in the dark at room temperature, according to the manufacturer’s recommendations. The stained cells were examined by BD FACSCalibur flow cytometer (BD Biosciences, California, USA) equipped with Cell Quest software (BD Biosciences).

### Senescence-associated β-galactosidase staining

The cells were plated in 6-well plates and then cultured overnight in complete medium in the presence or absence of IL-1β (10 ng/ml) for 24 h. Senescence-associated β-galactosidase (SA-β-gal) activity was detected with an SA-β-gal staining kit following the manufacturer’s protocol.

### Real-time PCR

Cells were cultured in 6-well plates, and total RNA was extracted using Trizol (beyotime) according to the manufacturer’s protocol. One thousand nanograms of total RNA was reverse-transcribed to first-strand complementary DNA (cDNA). The converted cDNA samples were amplified in triplicate by real-time PCR (Roche cobas z 480) in a final volume of 20 ml using SYBR Green. When evaluating the effect of a treatment, the expression level of each control was assigned an arbitrary value of 1, and the treated cells were evaluated as fold change over control and calculated as 2^-as a^. GAPDH was used as invariant housekeeping gene internal control. miRNA expression was normalized to U6 small nuclear RNA.

### Western blotting

Cells were lysed in RIPA with 1 mM phenylmethanesulfony fluoride (PMSF, Beyotime) and the protein content of the lysates was determined using a bicinchoninic acid (BCA) protein assay reagent (Pierce, Rockford, USA). Cell lysates were adjusted to equal equivalents of protein and then were applied to SDS epolyacrylamide gels for electrophoresis as described before [19].

### Statistical analysis

All experiments were performed independently at least three times. Data are expressed as mean ± standard deviation (SD). All analyses were performed using SPSS version 20.0 software (SPSS, Inc., Chicago, USA) and graphs were generated using GraphPad Prism 5 Software (GraphPad Software, San Diego, CA, USA). Statistical significance was assessed by one-way analysis of variance (ANOVA). *P* < 0.05 was considered statistically significant.

## Results

### Expression of MMP13, ADAMTS5, and COL2A1 in normal chondrocyte and OA chondrocyte

**Fig. 1:**
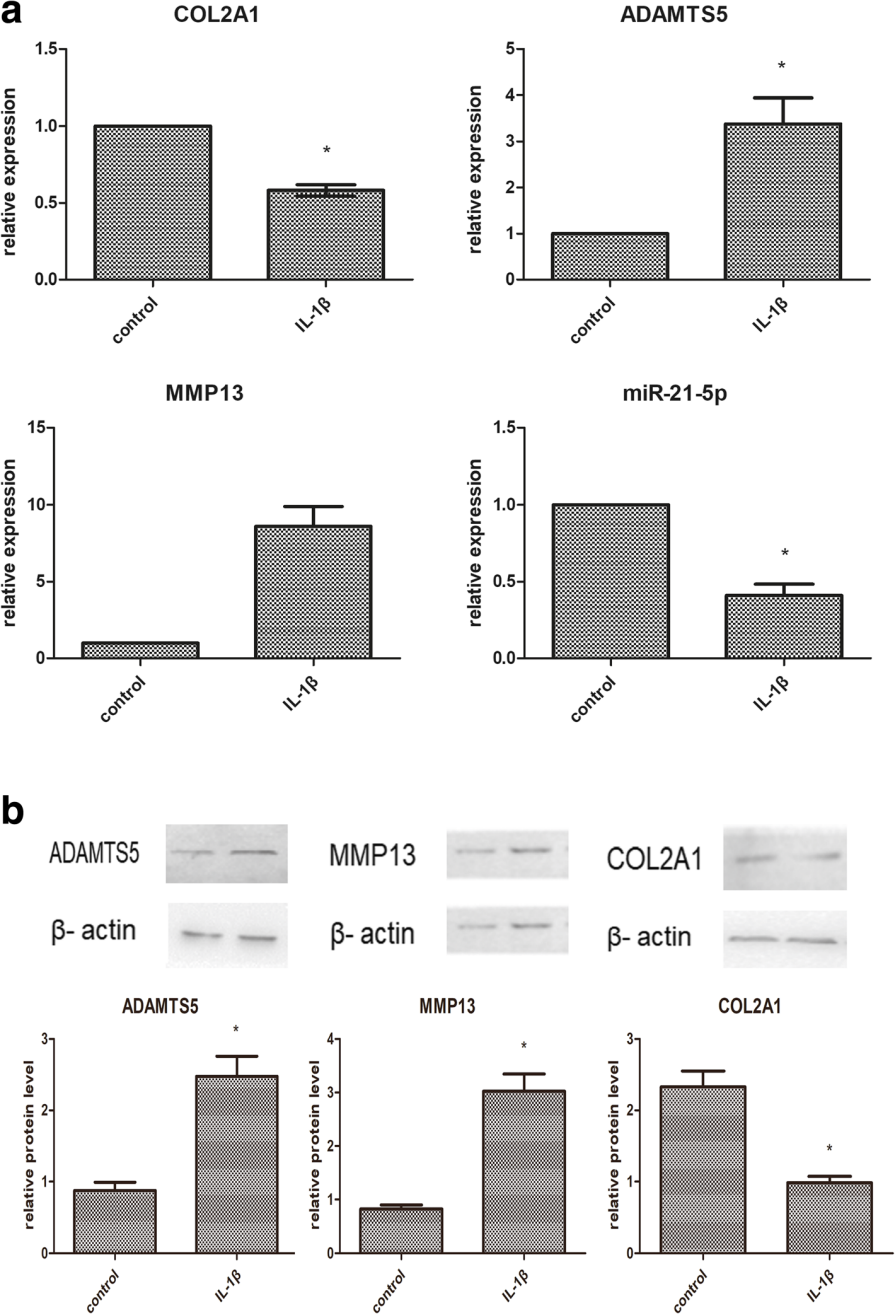
In vitro expression of chondrocyte degeneration by IL-1β stimulation. Normal chondrocytes were treated with IL-1β (10 ng/ml) for 24 h (**a**). qPCR detected the expression of COL2A1, ADAMTS5, MMP13 and miR-21-5p (**b**). Western blot analysis showing the expression of COL2A1, ADAMTS5, MMP13 in normal and IL-1β treated chondrocytes. β-actin expression and U6 were detected as an endogenous control. Quantification of the western blot data was detected by densitometric analysis.*P < 0.05 vs control group. ^#^*P <* 0.05 vs OA group

To verify that chondrocytes stimulated by IL-1β (10 ng/ml) demonstrate characteristics of normal and OA chondrocytes, we examined expression levels of ADAMTS5, MMP13, and COL2A1. In OA pathogenesis, COL2A1 is downregulated, while ADAMTS5 and MMP13 are upregulated. As shown in Fig. 1, the expression of ADAMTS5 and MMP13 were significantly increased in IL-1β stimulated chondrocytes, while the expression of COL2A1 was significantly decreased compared with normal chondrocytes. These results suggest that IL-1β-simulated normal articular chondrocytes can mimic OA chondrocytes. Therefore, we used IL-1β to induce in vitro conditions of OA chondrocytes.

According to the Fig. 1, we also found that miR-21-5p level was significantly decreased in OA chondrocytes, compared to the normal chondrocytes.

### Upregulation of miR-21-5p promotes COL2A1 synthesis and downregulates ADAMTS5 and MMP13 expressions

To determine whether miR-21-5p expression changes the OA progression, we transfected the OA chondrocytes with miR-21-5p mimic and inhibitor and detected the expression of COL2A1, ADAMTS5, and MMP13.

**Fig. 2:**
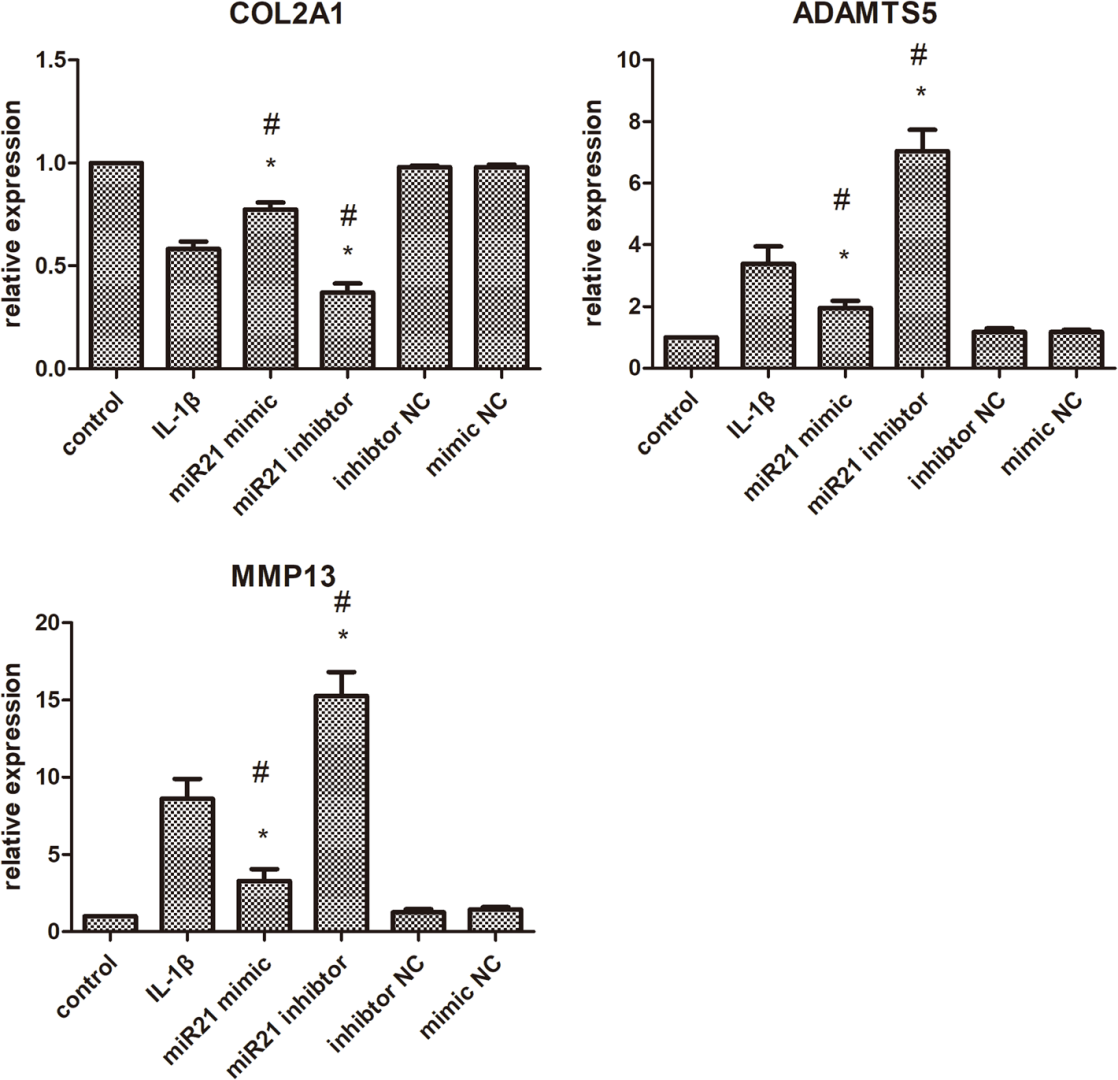
Upregulation of miR-21-5p promotes COL2A1 synthesis, downregulates ADAMTS5 and MMP13 expressions. qPCR detected the expression of COL2A1, ADAMTS5, MMP13. U6 expression was detected as an endogenous control. *P < 0.05 vs OA chondrocytes, ^#^*P* < 0.05 vs OA group

After transfections, we analyzed the expressions of COL2A1, ADAMTS-5, and MMP13 in OA chondrocytes. qRT-PCR assay showed that the expression of COL2A1 was increased in chondrocytes that were transfected with miR-21-5p mimic compared with mimic control, inhibitor control, or miR-21-5p inhibitor transfected cells (Fig. 2). As shown in Fig. 2, knockdown of miR-21-5p in OA chondrocytes strongly increased ADAMTS5 and MMP13 levels, whereas overexpression of miR-21-5p decreased ADAMTS-5 and MMP13 levels. Collectively, the data indicate that the upregulation of miR-21-5p could promote chondrocytes matrix synthesis and decrease ADAMTS5 and MMP13 levels.

### Upregulation of miR-21-5p promotes cell proliferation and reduces cell apoptosis

**Fig. 3:**
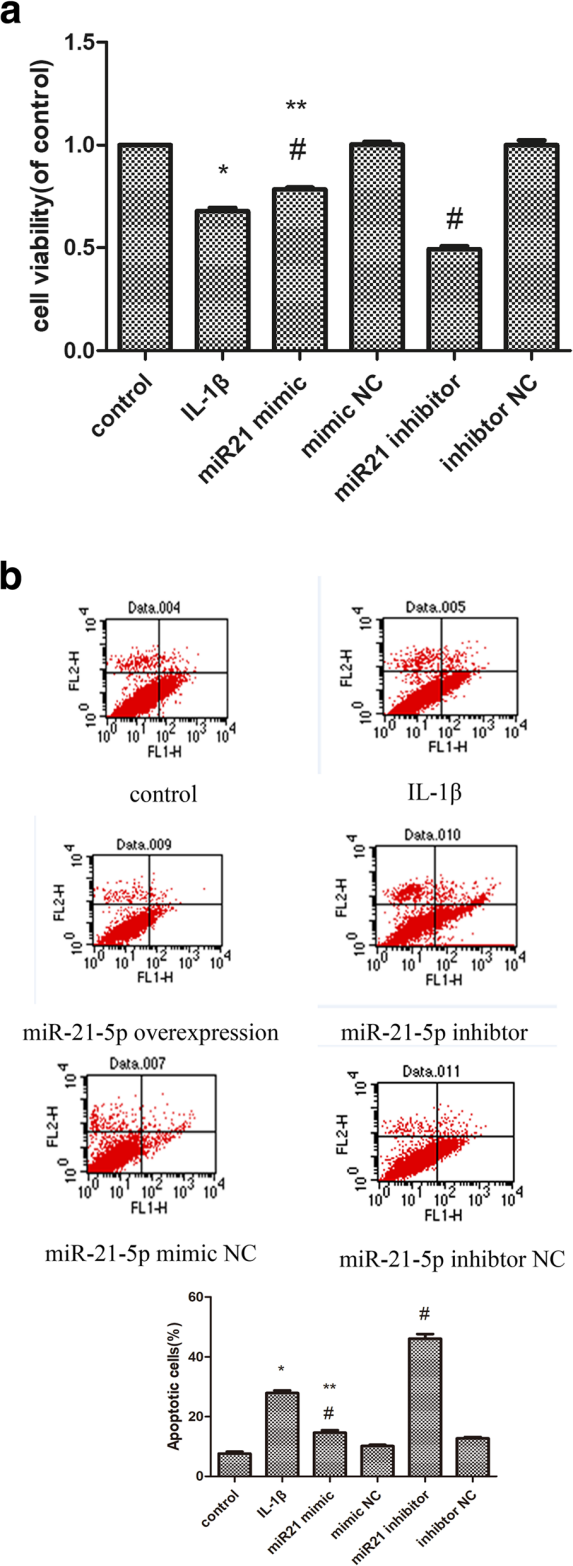
Effect of miR-21-5p on OA chondrocytes (**a**). CCK-8 assay was to quantify viable cells (**b**). the cells were subjected to FACS analysis to determine the cell apoptosis rate. *P < 0.05 compared with the normal group. ^#^*P* < 0.05 compared with the OA group.

In the Fig. 3a, we could find that the cell viability of OA chondrocytes was significantly lower than normal chondrocytes, while the cell apoptosis was obviously higher than normal chondrocytes.

At 48 h following miR-21-5p mimic transfection, OA chondrocytes viability was increased, compared to miR-21-5p inhibitor, as examined by CCK8 assay. The cell viability of IL-1β was 69.49% l5.27%, which decreased compared to that of normal cell, in contrast, the viability of cells treated with miR-21 mimic was 80.38% ± 2.09%.

With respect to chondrocytes apoptosis, a significant increase was shown in Fig. 3b in OA chondrocytes compared with normal controls (26.49% ± 4.48% vs. 5.13% ± 0.83%) (*P* < 0.05). The overexpression of miR-21-5p significantly decreased the expression of chondrocytes apoptosis, compared with miR-21-5p inhibitor (15.91% ± 1.36% vs.44.92% ± 3.25%) (*P* < 0.05).

### Senescent behavior

**Fig. 4:**
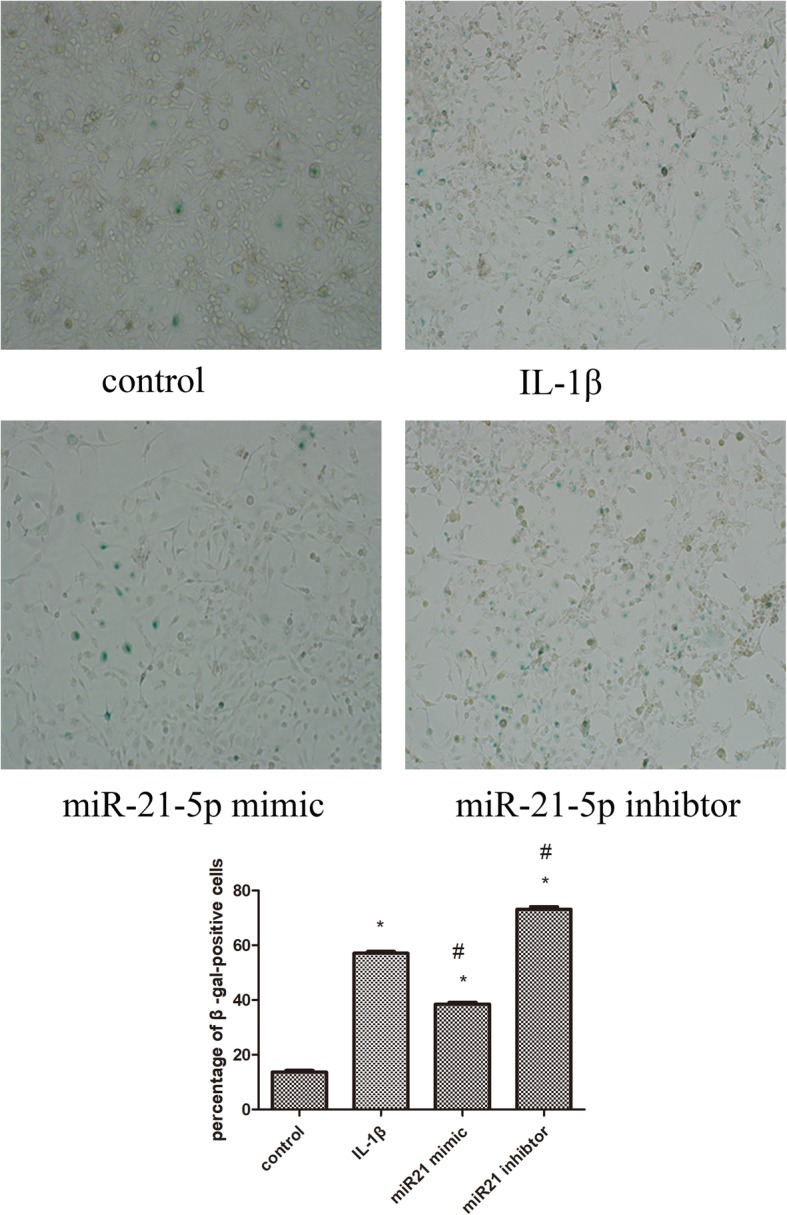
To identify senescent cells, chondrocytes were stained with SA β-gal, and observed under a light microscope (magnification × 100). Values represent the mean ± SD from three independent replicate experiments.*P < 0.05 compared with the normal group. ^#^*P* < 0.05 compared with the OA group

As shown in Fig. 4, OA chondrocytes exhibited senescent behavior as our previous study had confirmed [8], and in this study, we confirmed this result again.

When the cells were transfected with miR-21-5p mimic, SA-β-positive cells were 37.72% ± 3.78%, which were decreased compared to that of OA chondrocytes (*P* < 0.05). While SA-β-positive cells transfected with miR-21-5p inhibitor were higher than OA chondrocytes (74.17% ± 2.56%).

There were 15.49% ± 3.72% and 57.57% ± 2.84% SAβ-gal positive cells, respectively, for normal controls and OA group.

## Discussion

As the population worldwide ages, OA, which is the most common type of arthritis, is gradually becoming a serious health problem for individuals and a huge economic burden for the whole social health system. Articular cartilage has a limited capacity for self-repair because of its avascular nature and the low proliferation rate of chondrocytes [20]. Failure to reverse the disease process implies that a long-term disease management is definitely necessary. Developing therapeutic molecules that target chondrocytes and locally produced inflammatory factors within arthritic cartilage is an active area of investigation.

The miRNA family consists of single-chain, non-coding small RNA molecules that are usually 22–24 nucleotides in length [21]. MiRNAs are known as one class of small non-coding RNAs which have critical roles in regulation of a variety of vital processes such as angiogenesis, growth, invasive, metastasis, and differentiation. In recent years, some miRNAs exhibit an anti-apoptotic function, resulting in the stimulation of tumorigenesis. Silencing of miR-21 in glioblastoma cells could lead to inhibition of cell proliferation in vitro and could decrease tumor formation in vivo [22]. miR-21-5p induces tumor angiogenesis through inhibition of PETN and subsequent up regulation of HIF and VEGF levels [23]. MiR-21-5p also plays a protective role in myocardial apoptosis through PTEN/Akt signaling pathway [24].

IL-1β is one of the most prominent mediators of cartilage degradation and joint inflammation [25]. IL-1β induces a cascade of inflammatory and catabolic events in chondrocytes [8]. It also changes chondrocyte anabolism by suppressing the synthesis of proteoglycans and collagens and by enhancing the production of MMPs [26]. In our present study, the expression of miR-21-5p was downregulated by IL-1β stimulation of chondrocytes in vitro. These data suggest that IL-1β may be a mediator that is involved in the suppression of miR-21-5p in OA.

It is widely accepted that MMPs and ADAMTS play critical roles in cartilage extracellular matrix degradation during the pathogenesis of OA since they are responsible for the degradation of type II collagen and proteoglycans in articular cartilage [27, 28]. MMP overexpression can play an important role in the destruction of cartilage in OA. MMP-13 breaks down proteoglycans, such as aggrecan and collagen, in the extracellular matrix and is thought to be a major mechanism of cartilage destruction in OA [29]. The ADAMTS family is complex secreted proteins that have crucial and wide-ranging roles in tissue morphogenesis and pathophysiological remodeling, in inflammation and in vascular biology [28]. There is evidence that ADAMTS5 is considered as the primary aggrecanase responsible for the cleavage of aggrecan in the pathogenesis of the OA and are potential targets for therapeutic intervention in OA [30].

In the present study, we found the level of miR-21-5p was significantly lower in OA chondrocytes than that of normal chondrocytes. More importantly, miR-21-5p expression level was negatively correlated with cartilage degeneration. It has been showed that the deregulation of Col II, ADAMTS5, and MMP13 could be related with initiation and progression of OA. In addition, we verified that the upregulation of miR-21-5p in OA chondrocytes could improve the changes of Col II, ADAMTS5, and MMP13, reverse remodeling of the cartilage extracellular matrix. These findings indicate that miR-21-5p may be employed as a therapeutic biomarker for OA.

The ability of chondrocytes to remodel and repair cartilage ECM declines with aging, and in OA this is related to a decline in the anabolic activity of chondrocytes [31]. Our data not only changed Col II, MMP13, and ADAMTS5, but also altered OA chondrocytes apoptosis and senescence. In the previous study, we found that OA chondrocytes showed the increased cell apoptosis and senescent behavior [8].

The correlation between chondrocyte death and the generation and progression of OA is still controversial. Death of chondrocytes usually takes place in the terminal stages of OA progression. During this stage, the number of cells in highly fibrillated cartilage matrix is significantly decreased, indicating that the death of chondrocyte serves as a terminal event in OA [32]. Nevertheless, several studies claimed that chondrocyte death served as an initiating event [33].

Hence, better understanding of the underlying mechanisms of apoptosis could contribute to finding and developing new therapeutic platforms. Previous studies have suggested that miR-21 could play an essential role in modulating cell proliferation [34, 35]. In addition, the proliferation of OA chondrocytes was significantly enhanced when the cells were transfected with overexpression of miR-21-5p.

In OA cartilage, an association between decreased expression of miR-146a and increased expression of MMP-13 was observed [36]. The expression pattern was quite similar to that of miR-140-5p and miR-320, which has been shown to stimulate chondrogenesis in vitro [37, 38].

Targeting-expressed miRNAs are often disease-specific and do not play roles under normal physiological conditions in adult tissues or in quiescent cells [39]. On the basis of our findings, the development of miRNA-21-5p-based therapeutics for the treatment of OA is promising in the future. To explore potential application of miR-21 as a therapeutic agent, further study is needed to evaluate the effectiveness and safety of miR-21 in vivo. Different miRNAs may suggest a novel treatment modality. Based on the broad participation of miRNAs in OA, we supposed that different miRNAs may co-regulate OA-related genes and synergistically affect the associated pathological processes. Further work needs to be explored.

## Conclusion

MiR-21-5p might be a potential disease-modifying compound in OA, as it promotes hyaline cartilage production. These results provided that novel insights into the important function in OA pathological development.
