# Correction to: miR-21-5p protects IL-1β-induced human chondrocytes from degradation

**DOI:** 10.1186/s13018-020-01625-6

**Published:** 2020-03-10

**Authors:** Hai Zhu, Xin Yan, Meng Zhang, Feng Ji, Shouguo Wang

**Affiliations:** 1 Department of Orthopaedics, The Affiliated Huaian No.1 People’s Hospital of Nanjing Medical University, Huaian, Jiangsu Province China; 2 Department of Orthopaedics, Affiliated Hospital of Nantong University, Nantong, Jiangsu Province China; 3 Research Center of Clinical Medicine, Affiliated Hospital of Nantong University, Nantong, Jiangsu Province China

**Correction to: J Orthop Surg Res (2019) 14:118**

**https://doi.org/10.1186/s13018-019-1160-7**

Following publication of the original article [1], due to mistakes, the flow chart of miR-21 overexpression, miR-21 inhibitor and miR-21 mimic NC in Fig. 3, and the corresponding histogram need to be replaced. The senescent behavior of miR-21 mimic and miR-21 inhibitor in Fig. 4 will be replaced, accompanying the relative histogram.

**Fig. 3:**
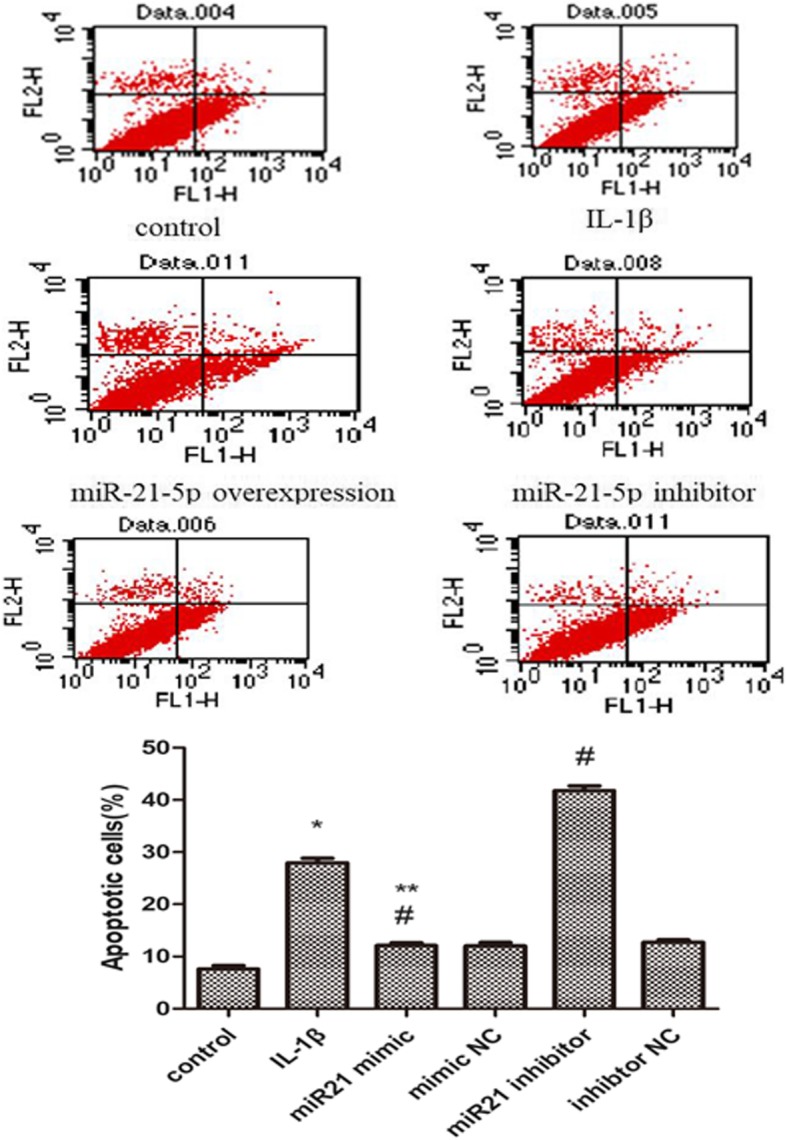
Effect of miR-21-5p on OA chondrocytes (**a**). CCK-8 assay was to quantify viable cells (**b**). the cells were subjected to FACS analysis to determine the cell apoptosis rate. *P < 0.05 compared with the normal group. ^#^*P* < 0.05 compared with the OA group

**Fig. 4:**
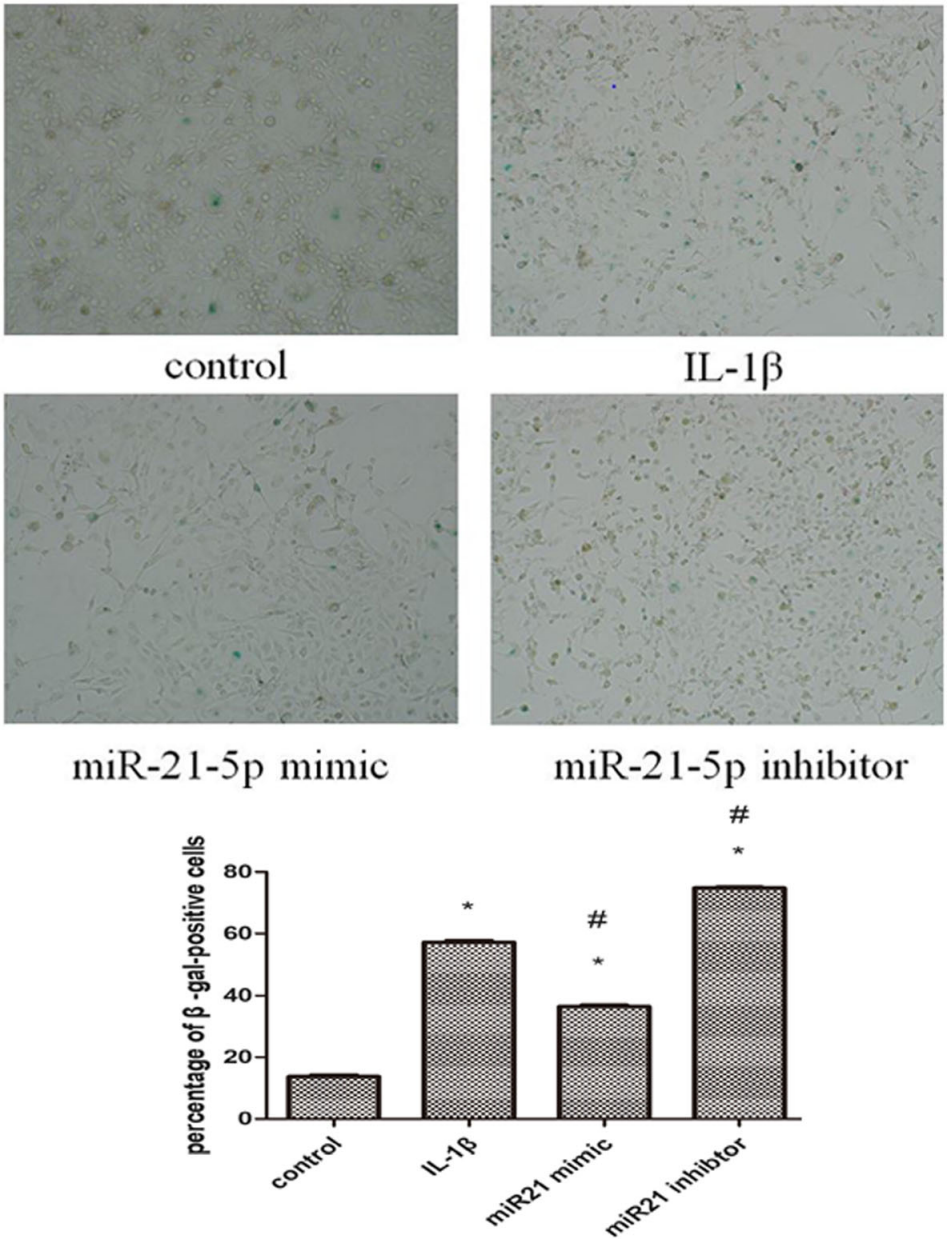
To identify senescent cells, chondrocytes were stained with SA β-gal, and observed under a light microscope (magnification × 100). Values represent the mean ± SD from three independent replicate experiments.*P < 0.05 compared with the normal group. ^#^*P* < 0.05 compared with the OA group
